# Factors Associated with E-Cigarette Escalation among High School Students: A Review of the Literature

**DOI:** 10.3390/ijerph181910067

**Published:** 2021-09-25

**Authors:** Michael Short, Adam Geoffrey Cole

**Affiliations:** Faculty of Health Sciences, Ontario Tech University, Oshawa, ON L1G 0C5, Canada; adam.cole@ontariotechu.ca

**Keywords:** escalation, vaping, E-cigarette, adolescent, frequency, intensity

## Abstract

Background: E-cigarette use has been identified as a behaviour of concern among adolescents, and ever and daily use among this population has increased recently. The purpose of this review was to summarize the relevant studies investigating the frequency and intensity of e-cigarette use in adolescents and the factors associated with these patterns of use. Methods: A scoping search of two databases was conducted to identify longitudinal studies examining escalating e-cigarette use among adolescents. Escalating e-cigarette use could refer to an increasing frequency or intensity of use over time. Articles were screened for relevance. Studies that met inclusion criteria were included for synthesis. Results: Five articles were included for synthesis. All five articles were longitudinal studies taking place in the United States between 2013 and 2017. Age, gender, cost of e-cigarettes, use of cigarettes, polysubstance use, and e-liquid nicotine concentration were associated with escalation of e-cigarette use. Conclusions: A paucity of information exists regarding the escalation of e-cigarette use among adolescents. Given the changing popularity of devices, additional updated evidence is needed to understand the factors associated with the escalation of e-cigarette use among adolescents, which can be used to inform local and national programs and policies.

## 1. Introduction

E-cigarettes, also known as vapes, are electronic devices designed to aerosolize a liquid which is then inhaled by the user [1]. These devices can deliver doses of sweetly flavored aerosol (often referred to as “vapor”), which frequently contains nicotine [2,3]. Despite the fact that many people believe that e-cigarettes are harmless, the aerosol contains propylene glycol, glycerin, aldehydes, and other toxins [2,4]. By virtue of the ingredients contained in the e-liquid, e-cigarettes have the propensity to cause a range of health issues including lung inflammation, irregular brain development and alteration of neurotransmitters such as dopamine [2,4]. Other evidence has found that using e-cigarettes is associated with higher odds of experiencing bronchitic symptoms [5]. The nicotine found in e-cigarettes can alter a variety of physiological functions, including blood pressure, which may lead to adverse health events such as strokes and myocardial infarctions [2]. These health concerns are especially worrisome for the adolescent population as their brain is still developing, and the addition of nicotine at this stage of development may make them more susceptible to addiction [2].

Along with the emerging health concerns of e-cigarette use, there is growing concern that e-cigarettes may appeal to adolescents, particularly those who would otherwise not engage in tobacco use [4]. Recent evidence indicates that e-cigarette use among adolescents is globally problematic [1,3,6]. Data from the National Youth Tobacco Survey (NYTS) illustrates that the prevalence of e-cigarette use among grade 12 students increased from 9.0% in 2004 to 13.7% in 2014 [7]. More recent data indicate that daily e-cigarette use among grade 12 and grade 10 students was 11.7% and 6.9%, respectively, in 2019 [8]. Furthermore, this study also reported that ever use of e-cigarettes increased from 34.0% to 40.5% among grade 12 students from 2018 to 2019 and increased from 28.6% to 36.4% among grade 10 students between 2018 and 2019 [8]. The concerning increasing prevalence of e-cigarette use is not exclusive to the United States. In a 2015 study of Irish youth, 24% of adolescents reported having used an e-cigarette in their life while 3.2% reported current use (defined as at least once a month) [9]. Furthermore, in 2017, up to 18% of adolescents in the United Kingdom had ever used e-cigarettes and 3% of adolescents reported daily use [10]. Lastly, in a study analyzing the trends of e-cigarette use among adolescents in Ontario, Canada, it was found that use of e-cigarettes increased from 7.6% in 2013 to 25.7% in 2018 [11]. 

With an increase in the prevalence of e-cigarette use among adolescents, it is imperative to understand the allure of these products to the adolescent population. Several studies have investigated the factors associated with the initiation of e-cigarette use [4,7,9,12,13]. Multiple studies have reported that gender, friend use, and perception of e-cigarettes, as well as previous cigarette use are associated with e-cigarette initiation [4,7,9,12,13]. However, it has yet to be seen if these factors are also associated with escalating e-cigarette use. 

While researchers have spent a significant amount of time studying e-cigarette initiation since the introduction of the devices, it appears as though less attention is being paid to the increase in frequency and intensity (both measures of escalation) of e-cigarette use. Frequency of e-cigarette use is typically expressed as the number of days in the last 30 days in which the participant has used an e-cigarette [14,15,16,17,18]. Intensity can be defined as the number of times a participant used their e-cigarette in a given day [15]. Together, frequency and intensity compose the metric for e-cigarette escalation, as an increase in either metric can mark an increase in use of e-cigarettes [15]. 

The Theory of Triadic Influence (TTI) has been used to address substance use behaviour in adolescents, being of specific use in the realm of cigarette smoking and escalation [19,20]. TTI frames substance use in the dimensions of proximal (attitudes, beliefs, and feelings of self-efficacy) and distal influences (intrapersonal and interpersonal issues stemming from the socio-cultural environment) [20]. The theory highlights the complexities surrounding substance use initiation and escalation. As such, a deep understanding of behaviours and influences at multiple levels must be obtained in order to exact the necessary policies and programs to tackle the complexities associated with use [19,20].

To our knowledge, there has yet to be a review of the literature investigating escalating use of e-cigarettes among adolescents. Therefore, the aim of this literature review was to search the literature and summarize the pertinent studies on the frequency and intensity of e-cigarette use and the factors associated with the increases in frequency and intensity of e-cigarette use. 

## 2. Materials and Methods

We conducted a search of the literature to summarize available evidence regarding escalating e-cigarette use among adolescents. Escalation was defined in two ways: (1) an increase in the intensity of using e-cigarettes (number of events of e-cigarette use per day), or (2) an increase in the frequency of using e-cigarettes (number of days in the past 30 days in which the participant had used e-cigarettes). 

### 2.1. Eligibility Criteria

Eligible articles met the following criteria: (1) published in English, (2) investigated an increase in frequency or intensity of e-cigarette use, (3) sample was adolescents aged 13 to 19 years old, to capture a sample of high school students, (4) longitudinal study design, and (5) published between January 2009 and October 2020. The longitudinal study design is a necessary criterion when investigating e-cigarette escalation. A study must be able to exhibit an increase of a participant’s escalation over time. Cross-sectional studies are insufficient for capturing escalation as they provide a prevalence of adolescents engaging in a particular level of use (either frequency or intensity), but do not provide insight into a change of behaviour over time. Methodologically, this is due to the single point in data collection associated with cross-sectional studies. The publication dates for eligible studies ranged from the time e-cigarettes were introduced into the market (1 January 2010) to when the search took place (25 October 2020). 

Studies were ineligible for inclusion if they were not published, peer-reviewed studies, as well as if they were pilot studies, letters to the editor, abstracts only, or cross-sectional studies, as these types of publications do not provide sufficient information on study methodology or results. Studies that were published in a language other than English (without English translation) were also excluded. Furthermore, studies that investigated escalating e-cigarette use among those aged over 19 years of age were excluded, as these studies may present associations that are not applicable to the adolescent populations, as were studies that investigated escalating combustible cigarette or cannabis use in the absence of e-cigarette use.

### 2.2. Search of the Literature

The initial search strategy was developed and used in MEDLINE and then adapted for use in CINAHL. Databases were chosen based on their breadth of coverage of the literature. The following terms were used in combination to search the literature for appropriate articles: students, adolescents, youth, high school, vaping, vape, e-cigarette, escalation, frequency, and intensity. EndNote was used to capture the results of the search, record the number of duplicates, and provide a method of organization for abstract and title screening. Duplicates were removed from the search results. Further articles were identified by datamining included articles for additional articles that may have evaded the search parameters. 

### 2.3. Study Selection

Studies were selected in a two-phase approach. First, one author (M.S.) screened the titles and abstracts of all relevant articles produced from the initial search of the literature against the inclusion criteria. This involved yes/no responses to whether studies were of the appropriate type, investigated the appropriate dependent variable, and was performed in an appropriate sample. The second phase of selection involved one author (M.S.) screening the full text of all remaining articles using the same checklist as in the first phase. If unsure about the inclusion of an article, an expert in the field (A.G.C.) was consulted regarding the applicability of the study.

### 2.4. Data Collection and Synthesis

The following data were collected from each relevant study when applicable: date of data collection, descriptive statistics of the sample, geographic location of the study, descriptions of the measure of e-cigarette use, and study findings. The study findings could include means and standard deviations (for descriptive statistics), prevalence, and odds ratios, or relative risks identifying the associations between demographic variables and escalation of vape use.

## 3. Results

### 3.1. Search Results

**Figure 1:**
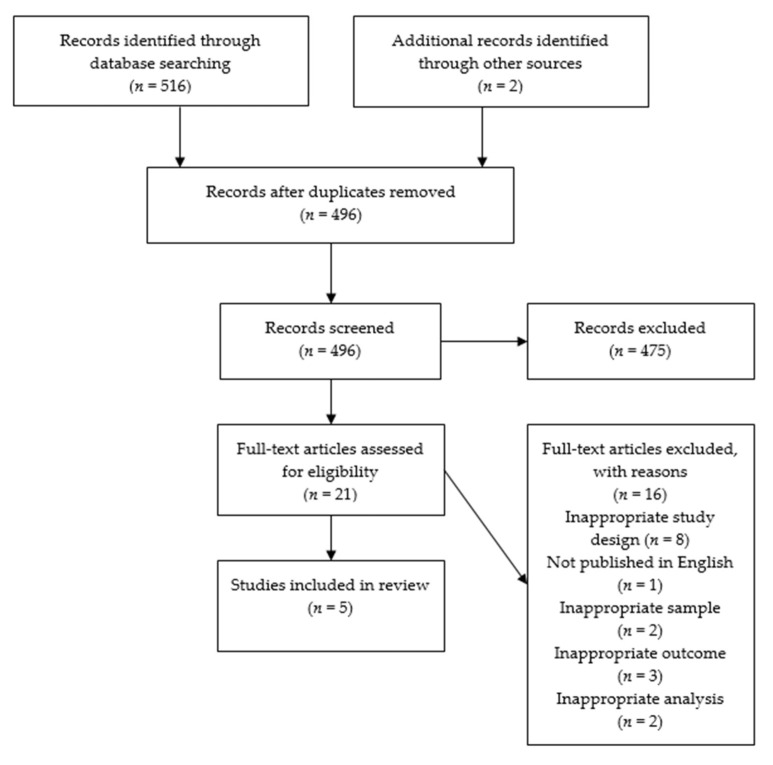
PRISMA diagram of search results.

As shown in Figure 1, we retrieved 516 articles from MEDLINE and CINAHL databases with two articles added from outside of the search parameters (mined from the reference list of included articles). Twenty-two duplicate articles were initially removed leaving 496 articles for abstract and title screening for eligibility. Of these, 21 articles were selected for full-text screening. Reasons for exclusion from full-text screening included the following: (1) ineligible population (i.e., young adults, college students), (2) cigarette use investigated rather than e-cigarette use, and (3) no measures of frequency or intensity of e-cigarette use. After full-text screening, five articles were selected for inclusion in the review. Studies failing full text screening did so for the following reasons: inappropriate study design (*n* = 8); published in a language other than English (*n* = 1); sample size composed of solely combustible cigarette smokers (*n* = 2); did not investigate escalation of e-cigarette use (*n* = 3); and did not include e-cigarettes in their analysis of smokeless tobacco use and cigarette smoking (*n* = 2).

### 3.2. Characteristics of Included Studies

All five studies included for review were longitudinal studies and were conducted in the United States [14,15,16,17,18]. All studies included a form of measurement that investigated either increasing frequency or intensity or an increase in both, representing an escalation of e-cigarette use. Sample sizes for the included studies ranged from 181 to 101,011.

### 3.3. Summary of Evidence

Characteristics for the five studies included in the synthesis of results are presented in Table 1.

The first longitudinal study collected data from adolescents attending three high schools or two middle schools in the United States (*n* = 340) [14]. E-cigarette frequency was assessed in both waves using the question, “How many days out of the last 30 did you use e-cigarettes?” Among the sample of ever e-cigarette users, the authors identified that frequency of use increased from 7.4 (± 9.6) days in the past 30 days in wave 1 (Fall 2013) to 10.4 (± 10.5) days in the past 30 days in wave 2 (Spring 2014) [14]. Many reasons for initiating e-cigarette use were predictive of an increase in frequency of their use, including (1) good flavors (41.8% of sample); (2) easy to hide from adults (12.9%); (3) low cost (10.0%); (4) ability to use e-cigarettes anywhere (20.9%); and (5) to quit smoking regular (combustible) cigarettes (5.9%). Low cost was found to be the strongest predictor of escalation of e-cigarette use in this population after adjusting for covariates (OR: 3.08; 95%CI: 1.34, 7.08). The authors also identified that a younger age of onset of e-cigarette use was associated with an increased use of e-cigarettes (OR: 2.86, 95%CI: 1.01, 8.13) [14].

A second longitudinal study included data from 181 students from ten high schools in Los Angeles, California. Frequency of e-cigarette use was measured by assessing the number of days out of the last 30 days in which students used e-cigarettes. The intensity of e-cigarette use was measured using the question, “On the days you vaped, how many times did you usually pick up your e-cigarette device to vape?” Participants had the following response options: 1 time, 2 times, 3–5 times, 6–9 times, 10–14 times, 15–20 times, or ≥20 times [15]. Of those who reported vaping nicotine, 28.9% reported vaping a low concentration (1–5 mg/mL), 19.3% reported vaping a medium concentration (6–17 mg/mL), and 11.6% reported a high concentration (18 mg/mL or more) [15]. Although the authors identified a positive association between baseline nicotine concentration and baseline frequency of use (OR = 1.65; 95%C.I.:1.09, 2.51), there was no association between baseline e-cigarette concentration and frequency, or intensity of e-cigarette use at follow up [15].

The third longitudinal study compared the trajectory of cannabis-containing e-cigarette use with the trajectory of nicotine-containing e-cigarette use among high school students [16]. Vaping frequency was assessed by the following two questions: (1) “In the last 30 days, how many total days have you used an electronic cigarette with nicotine (e-cigs, personal vaporizer, (PV))?”; and (2) “In the last 30 days, how many total days have you used an electronic device to vape THC (tetrahydrocannabinol) or hash oil (liquid pot, cannabis oil, weed pen, PAX Era (PAX Labs, Inc., San Francisco, CA, United States))?” Five trajectories were identified for nicotine e-cigarette use. These trajectories were no use (67.6% of sample), infrequent use (17.0% of sample), moderate use (5.0% of sample), young adult-onset frequent use (6.4% of sample), and adolescent-onset escalating frequent use (3.9% of sample). Participants in the “Adolescent-Onset” group saw the average frequency of use increase from an average of 7.38 days in the past 30-days in adolescence to 21.49 days of use in young adulthood [16]. Males were shown to be at greater odds of belonging to the adolescent-onset escalating frequency trajectory when compared to females (adjusted Odds Ratio (aOR): 2.88, 95%CI: 1.03, 3.66) [16]. 

The fourth longitudinal study determined the developmental trajectory of e-cigarette use from early adolescence to late adolescence. E-cigarette use was measured using the single question, “On how many occasions (if any) did you use an electronic cigarette or e-cigarette (vape pen) during the last 6 months?” Answers were grouped in seven categories, labelled as never, 1 or 2 times, 3–5 times, 6–9 times, 10–19 times, 20–39 times, or 40 or more times. The authors identified three e-cigarette use trajectory groups: never users (66.6% of the sample), low and increasing users (20.1% of the sample), and high and increasing users (13.3% of the sample) [17]. Therefore, 33.4% of the sample escalated their e-cigarette use. This study determined that low e-cigarette use in early adolescence may serve as a risk factor for escalated use later on [17]. 

The last study investigated the patterns of use of e-cigarettes and their association with other substances in grade 8 students at 11 middle schools who were followed up in grade 9 in the subsequent year. At baseline, the average student reporting escalating e-cigarette use reported using an e-cigarette on 14 days out of the past 30 days and escalated to using e-cigarettes every day for the past 30 days at subsequent follow-up. The authors identified two e-cigarette use trajectory groups: non-users or low-frequency users (94.9% of the sample), and current and accelerating users (5.1% of the sample) [18]. Students who reported e-cigarette use were also more likely to use other substances (OR: 15.97, 95%CI: 7.72, 33.05) [18].

## 4. Discussion

Escalating e-cigarette use was observed in all five studies [14,15,16,17,18]. The studies ranged in a variety of analytic methods to identify escalating patterns of e-cigarette use. Only one study reported recent data that aligned with the rapid increase in popularity of high nicotine devices in the United States [16]. From the five articles included in this review, it is apparent that an increase in e-cigarette frequency has occurred, whereas an increase in intensity is less apparent due to the lack of reporting on this measure. Only one study included a measure for intensity among the included studies [15]. Frequency was much more commonly investigated, being a metric in all five of the included studies [14,15,16,17,18]. 

While frequency can provide insight into escalating e-cigarette use, it may be necessary to include both components, frequency and intensity, in future research to better grasp the true e-cigarette escalation patterns occurring in the adolescent population. For this to occur properly, standard valid measures of e-cigarette use frequency and intensity are needed. Researchers and policy makers may need to take into account the fact that adolescents may be using e-cigarettes in greater quantities sporadically throughout the month (i.e., in the form of intensity rather than frequency), similar to the concept of binge drinking observed in post-secondary students. Intensity can provide more detailed nuance into the study of escalation but also uncover additional issues with e-cigarette use. For example, an adolescent who is using e-cigarettes for each day in the last 30 days may be perceived as not being able to escalate their use further. However, if a study includes a metric to capture intensity, it is possible to capture escalation even at this high level of use (i.e., escalating from once per day in each of the last 30 days, to more than once per day in each of the last 30 days). This sort of concentrated behaviour was reported in a small group of adolescents who used e-cigarettes in social settings, such as at parties [21,22].

The result of this scoping review highlights a paucity of information regarding the escalation of e-cigarette use among adolescents. Only five articles were identified as capturing escalation among adolescents, with all five located in the United States. There is a need to perform similar studies across the globe to better understand the impact of e-cigarette escalation in adolescents. Without such information, it will be difficult to inform policy and design prevention programs which would assist in keeping adolescents around the world from engaging in risky health behaviours. The lack of available information is emphasized by the finding that only one study reported on the intensity of e-cigarette use. This is an important metric for escalation that requires more attention.

Across the included studies, few explored the sociodemographic or behavioural risk factors associated with e-cigarette escalation. Risk factors that were associated with escalating use of e-cigarettes among adolescents included comparative cost of e-cigarettes [14], gender [16], age [14], use of cigarettes [14,16], polysubstance use [16], and higher nicotine concentrations of e-liquid [15]. It is clear that research to-date has focused on proximal risk factors of e-cigarette escalation and has not examined the influence of more distal factors such as peer and parental e-cigarette use or local and national e-cigarette policies. Additional evidence is necessary to fully understand risk factors for e-cigarette escalation, particularly using more recent data, as devices have changed, and in other jurisdictions outside of the United States. Such knowledge could help inform the content and delivery of school- and community-based prevention and cessation programs. For example, it may be useful to educate students about the addictiveness of e-cigarettes and provide cessation resources at higher frequencies in order to prevent e-cigarette escalation [19]. Furthermore, evidence from other jurisdictions can reveal how different policy environments may influence the e-cigarette use patterns of youth.

While the evidence to-date has important implications for practice, due to the homogeneity in location of the studies identified, these implications may not be generalizable outside of the United States. Yet, it is clear that e-cigarette use has increased in popularity among adolescents, and some adolescents are escalating their use of e-cigarettes, increasing the risk of nicotine addiction and other adverse health effects. This concerning trend of e-cigarette use must be intervened upon to ensure the health of adolescents. The school environment is a common setting for intervention, because a range of students from diverse socioeconomic backgrounds attend for a large portion of the day. Effective school-based prevention and cessation programs include components that address a combination of social competency and social influence problems that are known to contribute to substance use initiation and escalation, such as self-esteem issues, dealing with peer pressure, and decision-making solutions [23]. Given evidence that polysubstance use is associated with e-cigarette escalation, school-based prevention programs should address multiple substances [24]. 

### Strengths and Limitations

Our review has several strengths. As far as we are aware, this is the first study that has collated the data relevant to the escalation of e-cigarette use in adolescents available in the literature. Furthermore, the study used a comprehensive search strategy to identify all relevant articles. Lastly, this study had a broad inclusion date, ranging from the January 2000 to October 2020.

This study was not without limitations. First, only articles published in English were included in the review. There may be applicable articles printed in other languages. Second, as this was a review of the literature, studies included were not critically appraised for methodological quality. Therefore, the results presented here should be interpreted with caution. Furthermore, articles were only reviewed by a single reviewer. While caution was made to include as many articles as possible that fit the criteria, it does leave open the possibility that an article was erroneously rejected from inclusion. Lastly, as only two databases were searched as saturation appeared to have occurred, there is a chance that other articles were unintentionally excluded. 

## 5. Conclusions

Despite the rising popularity of e-cigarette use, there is a paucity of research examining e-cigarette escalation and factors associated with escalation among adolescents. Evidence was exclusively from the United States, showcasing a need for international data on this topic. Additional knowledge of risk factors for e-cigarette use escalation is warranted to inform the design of adolescent e-cigarette use prevention and cessation activities. Due to increasing public health concerns, multiple interventions may be required to mitigate the increasing trend of escalating e-cigarette use.

## Figures and Tables

**Table 1:** Characteristics and results of included studies.

Authors	Sample Size, Location, and Year	Mean Age, and Gender	Methodology	Results
Bold, K.W., Kong, G., Cavallo, D.A., Camenga, D.R., and Krishnan-Sarin, S. [14]	340 students who reported previous e-cigarette useUnited States2013–2014	15.6 (±1.2) years of age52.6% female	Two waves of data collection (Fall 2013 and Spring 2014) assessing e-cigarette use frequency using the question, “How many days out of the last 30 days did you use e-cigarettes?”	Mean frequency of use increased from 7.4 (±9.6) days at wave 1 to 10.4 (±10.5) days at wave 2.Appeal of e-cigarettes to youth may be the ability to use e-cigarettes anywhere and the relatively low cost.Younger age (B = 10.19, SE = 3.30, ß= 0.24, *p* = 0.003) and use of traditional cigarettes (B = 4.86, SE = 1.94, ß = 0.23, *p* = 0.01) were associated with an increase in frequency of e-cigarette use
Goldenson, N., Leventhal, A.M., Stone, M.D., McConnell, R.S., and Barrington-Trimis, J.L. [15]	181 grade 10 studentsUnited States2013–2015	16.1 (±0.4) years of age53.0% male	Past 30-day use of e-cigarettes measured by frequency and intensity across five waves from Fall 2013 school semester to the Fall 2015 school semester.Frequency assessed by capturing number of days out of the past 30 days in which participants used e-cigarettes. Answers were grouped as no use (0 days), infrequent use (1 or 2 days), and frequent use (≥3 days).Intensity was assessed using the two questions: (1) “On the days you vaped, how many times did you usually pick up your e-cigarette to vape?” (responses: 1 time, 2 times, 3–5 times, 6–9 times, 10–14 times, 15–20, or ≥20 times); and (2) Each time you picked up your e-cigarette to vape, how many puffs did you usually take before putting it away?” (responses: 0 puffs, 1 puff, 2 puffs, 3–5 puffs, 6–9 puffs, 10–14 puffs, 15–20 puffs, or ≥20 puffs).	At baseline, 7.2% (*n* = 235) reported past 30-day vaping.Of an analytical sample of 181 students, 59.7% reported vaping a solution with nicotine in past 30-days, 28.7% vaped low concentration nicotine, 19.3% used medium concentration, and 11.6% used high concentration.After adjusting for covariates, for each 1-level increase in baseline nicotine concentrations, the odds of being a frequent 30-day vaper increased 1.65 times when compared to non-vapers at follow-up (OR: 1.65; 95%CI: 1.09, 2.51)
Lanza, H.I., Barrington-Trimis, J.L., McConnell, R., Cho, J. [16]	3322 high school studentsUnited States2013–2015	16.5 (±0.4) years of age53.5% female	Using data from waves 5–9 (when information regarding e-cigarette use was included in questionnaire) to create a trajectory of nicotine e-cigarette use.Nicotine vaping frequency assessed by the single question: “In the last 30 days, how many total days have you used an electronic cigarette, with nicotine (e-cigs, personal vaporizer, PV (personal vaporizer))?” Responses were coded into 0, 2, 4, 8, 15, 25, or 30 days.	Number of participants reporting any nicotine vaping in the past 30 days ranged from 4.4% in wave 7 to 7.5% in wave 8, before increasing to 22.0% in wave 9.Five trajectories identified:No use (*n* = 2246; 67.6%)Infrequent use (*n* = 566; 27.0%)Moderate use (*n* = 167; 5.0%)Young adult-onset frequent use (*n* = 213; 6.4%)Adolescent-onset escalating frequent use (*n* = 131; 3.9%)Highest increase in frequency of e-cigarette use occurred in the group of adolescent onset frequent users. This group was also associated with the cannabis vaping frequent user group. Cannabis and nicotine vaping may have similar underlying risk processes.
Park, E., Livingston, J.A., Wang, W., Kwon, M., Eiden, R.D., and Chang, Y. [17]	801 adolescentsUnited StatesYear not reported	14.14 (±1.37) years of age57% female	Data collected across five waves was used to create trajectories of e-cigarette use.E-cigarette use was assessed by a single question: “On how many occasions (if any) did you use an electronic cigarette or e-cigarette (vape pen) during the last 6 months?”. Response options included the following: Never, 1 or 2 times, 3–5 times, 6–9 times, 10–19 times, 20–39 times, or 40 or more times.	A total of 43.4% of the sample had tried e-cigarettes at least once before.Average age of onset for e-cigarette use was 14.1 (±0.5) years.Membership of trajectory groups:Never use (66.6%)Low and increasing (20.1%)High and increasing (13.3%)Even low frequency of use in early adolescence may be a risk factor for continuing and/or increasing use of e-cigarettes.
Westling, E., Rusby, J.C., Crowley, R., and Light, J.M. [18]	1130 adolescentsUnited States2014–2016	14.4 (±0.5) years of age53% female	E-cigarette use frequency was captured by two questions: 1. “In your whole life, how many different times have you ever smoked an e-cigarette (‘vape pen’) or an e-hookah, even a puff?”; and 2. “In the last 30 days, on how many days would you say you have smoked an e-cigarette (‘vape pen’) or an e-hookah, even a puff?”	Average e-cigarette use frequency was 14 days out of the last 30 days at baseline and increased to 30 days out of the past 30 days at follow up.A total of 1035 students (94.9%) were classified in the trajectory labelled “non or low users of e-cigarettes”.A total of 56 students (5.1%) were members of the “Accelerators” class, which denoted accelerating users of e-cigarettes.

B = unstandardized Beta value; β = standardized Beta value; SE = Standard Error; OR = Odds Ratio; 95%CI = 95% Confidence Intervals; *p* = probability value.
